# Relative Mortality among Criminals in Norway and the Relation to Drug and Alcohol Related Offenses

**DOI:** 10.1371/journal.pone.0078893

**Published:** 2013-11-06

**Authors:** Torbjørn Skardhamar, Vegard Skirbekk

**Affiliations:** 1 Senior researcher, Statistics Norway, Oslo, Norway; 2 Project Leader, Age & Cohort Change Program (ACC), International Institute for Applied Systems Analysis (IIASA), Laxenburg, Austria; University of California, United States of America

## Abstract

**Background:**

Registered offenders are known to have a higher mortality rate, but given the high proportion of offenders with drug-addiction, particularly among offenders with a custodial sentence, higher mortality is expected. While the level of overall mortality compared to the non-criminal population is of interest in itself, we also estimate the risk of death by criminal records related to substance abuse and other types of criminal acts, and separate between those who receive a prison sentence or not.

**Methods:**

Age-adjusted relative risks of death for 2000–2008 were studied in a population based dataset. Our dataset comprise the total Norwegian population of 2.9 million individuals aged 15–69 years old in 1999, of whom 10% had a criminal record in the 1992–1999 period.

**Results:**

Individuals with a criminal record have twice the relative risk (RR) of death of the control group (non-offenders). Males with a record of use/possession of drugs and a prison record have an 11.9 RR (females, 15.6); males with a drug record but no prison record have a 6.9 RR (females 10.5). Males imprisoned for driving under the influence of substances have a 4.4 RR (females 5.6); males with a record of driving under the influence but no prison sentence have a 3.2 RR (females 6.5). Other male offenders with a prison record have a 2.8 RR (females 3.7); other male offenders with no prison record have a 1.7 RR (females 2.3).

**Conclusion:**

Significantly higher mortality was found for people with a criminal record, also for those without any record of drug use. Mortality is much higher for those convicted of substance-related crimes: more so for drug- than for alcohol-related crimes and for women.

## Introduction

In this paper, we assess whether mortality is higher among criminal offenders, and to which extent this is explained by substance use. Although earlier studies have considered cause-of death, particularly focusing on drug overdose, there has been a lack of focus on studies that consider the type of criminal conviction (imprisonment or not) and whether the crime would suggest substance use (whether the crime was related to alcohol or drugs).

We provide evidence on elevated mortality among offenders with a criminal record, adjusting for age and socio-economic characteristics. Higher mortality in this group might not be surprising in itself given the large proportion of drug addicts, and particularly injecting drug users, 20 in this group. We study the all-cause relative mortality of people with a criminal record, and seek to provide an estimate of to what extent the excess mortality among offenders is driven by the drug-addicts in the sample, and whether also other offenders have an elevated mortality. We propose to use police records as a crude measure of drug addiction; given that particularly use- and possession of drugs is prohibited in Norway and the police records include *all* recorded offences (not only the principal offence), offenders with drug-addictions are likely to (also) have at least one drug offence record. In addition, our use of Norwegian administrative registers is likely to reduce problems that many earlier studies are subject to, in terms of non-representative samples, lack of national coverage or a relatively low sample size.

Estimates of the increased mortality from previous studies vary widely across settings and type of sample; and not enough is known on the causes of these differences. Previous studies have tended not to distinguish between drug-use and other life style risks. Some studies include information on causes of death, such as overdose from injections [1]–[10]. However, the recorded cause of death might often accurately reflect the effect of substance abuse as substance abuse can raise the mortality risk also from other causes.

Elevated mortality levels among former prison inmates who use drugs have been found in several studies, and has often been linked to overdose among opiate users [3], [4], [6], [7], [9], [11]–[14]. A study of Norwegians convicted for drug use (N = 1112) find that they have a significantly higher mortality risk than the overall population [15]. The risk of overdose among opiate users, particularly after a period of non-use such as a prison term, exacerbates the risk of death [2], [12], [15]–[17]. Ex-prisoners’ elevated risk of death has also been found to be directly or indirectly related to alcohol addiction, including violent death, liver disease and liver cancer [18]. One study found that people caught for driving under the influence of traffic-hazardous medications alone or in combination with alcohol had an increased risk of death in the six-year follow-up period after apprehension [19].

Incorrect classification or multiple causes of death can be a concern for populations addicted to alcohol and drugs [20]–[22]. E.g., Swedish data shows that chronic alcohol addictions are associated with increased risk of causes of death that are not directly related to alcohol [22]. There is evidence of an increased risk of deaths through accidents or being victim to murder among criminals [5], [6]. Cannabis users have higher risk of traffic accidents, and amphetamine/ecstasy users have a greater risk of hyperthermia and circulatory diseases [16]. Substance use has been found to be linked to serious depression and anxiety disorders, which in turn can raise suicide risk [23], [24]. Norwegians convicts, including those who are driving under the influence of alcohol, tend to have relatively low education, income, and parental status, particularly for women [25], and low socio-economic status tend to relate to higher mortality [26]. Studies have found that prisoners have a relatively high prevalence of hepatitis B/C virus, HIV/AIDS, tuberculosis and diabetes [5], [6], [12], [16], [18], [25], [27]–[29].

### Police records as indicators of substance abuse

While addiction to drugs is related to a range of criminal behaviours, [23], [30], [31] a large proportion of all crimes are more directly drug-related [32]. Thus, many people with excessive illegal drug use will be arrested at some point, although their principal offence might not be directly related to substance abuse. However, the Norwegian police are instructed to record all offences that could be included in an indictment. Thus, use- and possession of illegal drugs is likely to be recorded also in cases where it is not the principal offence. Use- and possession of drugs are also to a large extent related to the police’s stop-and-search practices, and drug users who spend much time in public areas are particularly at risk of being caught for such crimes sooner or later. It has also been argued that these offenders are largely drug-users themselves, being in possession of drugs for their own use or small-scale dealing to finance their own consumption [33]–[35]. For these reasons, we suggest that police records for use- and possession of illegal drugs is a strong indicator of substance abuse. It is likely that the majority of those with a high consumption of illegal drugs (especially those using hard drugs at the street level) over time have a very high probability of being caught at least once. This is also a group of persons where a particularly high mortality would be expected.

Clearly, some registered offenders will not have a record of drug offences even if they are drug users. They are likely to contribute to a higher mortality in the general offender group. On the other hand, some persons with substance abuse problems will not ever get a police record at all, and in our study, these will then contribute to the normal population. In addition, many with a moderate or infrequent consumption of e.g. cannabis will get such a police record even if their life style do not necessarily put them at elevated risk of death. Thus, we do not claim to make a rigid control for drug use, but we argue that our measure make it possible to a large extent separate the most heavy drug users from the remaining offender population. The remaining offender population will include some persons with substance abuse, but so does the non-offender population. Although our measures are imperfect, we suggest that the difference between the non-offender population and the non-drug-offenders is meaningful.

Further, individuals imprisoned with substance addiction problems are offered free drug rehabilitation programs in Norway. These programs aim to minimize drug use and are an integral part of the criminal care system which aims to help offenders to a normal life. There are drug-rehabilitation centres with 13 (out of 43) prisons in Norway and substance abusing inmates are offered such services [36]. One commonly used treatment option is the use of Opioid substitution treatment. Such programs were gradually introduced in the 1990s in Norway (by 2012 there were 7038 on such treatment) and they are likely to improve health outcomes of those who participated [37]. To the extent that such programs are successful in lowering mortality among substance abusing prison inmates, this would imply that our estimated mortality for this group of drug users is biased downwards and may represent a lower bound of what the actual mortality would have been in the absence of such treatments.

We also study persons with a police record for driving while being under influence of substances. While most of these persons are under the influence of alcohol, the influence of other substances is recorded using the same code in the crime statistics. A Norwegian study of blood samples from traffic controls reported that drugs were the primary suspicion in 40 percent of samples, but that drugs were detected in more than 70 percent of them [38]. Thus, a record of driving while intoxicated is an additional indicator of substance abuse, which might also be related to alcohol problems. The drink-drive limit in Norway was .5‰ up to year 1th January 2001, when it was lowered to .2‰. Thus, in the period relevant for our study, the drink-drive limit was similar to most European countries today [39]. One study of 12000 randomly selected drivers in Norway found that alcohol was present in 0.4% of the blood/urine samples taken [40]. Although the prevalence of drunk driving is high, and the proportion caught are relatively low, it is reasonable to suggest that most of those apprehended did not just happen to be caught on that single occasion, as the probability of arrest rises in proportion to the frequency the person is drunk driving. Thus, the prevalence of driving under the influence, although representing a sub-sample of all people with alcohol dependence, is also an important indicator of alcohol related problems. Thus, we argue that a police record of driving while intoxicated is a combined indicator of substance use and/or alcohol related problems. Although this measure will include groups without substance use that necessarily increase risk of death, this measure clearly separate some important groups with elevated death risk from the remaining offender population.

In sum, while police records on possession and use of illegal drugs, or police records of driving while intoxicated are far from perfect measures of drug addiction, it makes it to a large extent possible separate some offender groups with elevated mortality related to obvious life-style risks. Any elevated mortality in the remaining offender group is thus less likely to be related to these causes.

## Materials and Methods

The sample data for this study is defined from the Norwegian population registry as the total population of all persons aged 15–69 residents in Norway by the 31stof December 1999. We exclude people with an immigration background in order to have information on their parents’ educational level (N =  2.9 million persons of both sexes). Every resident has a unique national ID number used both by the various administrative registries and the police registration system. When data are sent to Statistics Norway they can also be linked at the individual level and used for research. We observe the death risks for the 2000–2008 period for those with a record of criminal activity during 1992–1999 (distinguishing between drug-related crimes, alcohol-related crimes and other crimes). Information on social background is taken from the National Educational Database, which contains a standard measure of the highest educational level of the mother and father when the focal individual was aged 16 (coding corresponds to ISCED97 level one, combining codes 0,1 and 3,4 and 5,6).

The information gathered from the police registration system includes all solved cases where the perpetrator has been identified by the police and where there is a “legal decision” against the offender [41]. As a result, these criminal records, although they often imply that there was a conviction, include people who were not convicted but received a prosecution waiver, the case was dropped because the suspect was under the age of criminal responsibility, or there was doubt about the suspect being of sound mind. Note that this implies that we capture also criminal records also persons under the age of criminal responsibility, although they do not get a conviction. Even where the person is sentenced for more than one offence, most studies using conviction data only have information on the principal offence. For example, less serious crimes like use and possession of drugs are often concealed by a more serious principal offence. One advantage of the Norwegian data is that every registered offence and every sanction received is included.

We categorise persons based on prevalence of alcohol- and drug-related offences. Naturally, the same person may commit a large number of offences and receive multiple kinds of sanctions over a given period of time. One alcohol- or drug-related offence might be accidental, but two or more is more likely to indicate a more systematic user. As drug-use is less prevalent and often considered more serious than alcohol, we include persons with one drug offence as a separate category. We use the entire history of registered offences and convictions in the period 1992 through 1999 and create categories based on a person having an official record of 1) committing *exactly one* offence related to use and possession of drugs, 2) committing *at least two* offences related to use and possession of drugs, 3) committing *at least two* offence related to driving* under the influence of alcohol, 3) having at least one registered offence of other kinds. (*Those under age 18 is not eligible for a driving license and thus are unlikely to have a record of driving while under the influence of alcohol. We have repeated the analysis including only those from age 20. This hardly affected the results at all.) We also split these categories based on a person having received at least one unconditional custodial sentence. Those with a criminal record that includes both alcohol- and drugs-related offences are counted under the drugs category. Drug smuggling is not included in this category.

The data in this analysis are based on national administrative registers and are not open to the public. The data is used within the general regulations that apply to Statistics Norway's work, and the ethics approval is obtained from the Data Protection Official. The project has formally been approved by the Statistics Norway's Data protection official, by letter 26. August 2011, according to Personal Data Act §8d, §9 and the Norwegian Statistical Act §1-1 and §3-1. The uses of Norwegian registry data is regulated by the Statistical Act and do not require consent from the participants if the project is approved by the Data Protection Official or the Norwegian Data Protection Authority. The data used was fully anonymized to ensure the protection of personal data.

We use all-cause mortality as our dependent variable. Information on causes of deaths was not available for this study. We estimate age-adjusted relative risk to investigate whether mortality varies by type of criminal background. The estimation is done using the modified Poisson approach with robust standard errors as implemented in SAS PROC GENMOD [42]. First, we estimate the relative risk of offenders versus non-offenders. Second, we control for social background to assess the explanatory factor of social inequality. Third, we split the estimates by whether or not the offenders were given a custodial sentence. Fourth, we split the estimates according to whether the offences were alcohol- or drug-related.

Women generally have a higher life expectancy than men, and while the offender population consists mainly of men, the female offender group is likely to be an even more strongly selected group than the male offenders. For this reason, we estimate separate models for men and women.

## Results

Table 1 shows the distribution of criminal records for the population studied and the proportion of deaths in each group. A full 10% (285 520 individuals) of the observational sample were registered for any offence in this period, and 1.8% (52691 individuals) had committed addiction-related crimes. Addiction-related crimes constitute 62% of all those with a prison record (drugs: 23%, alcohol: 39%) and 11% of those with a police record but not imprisoned (drugs: 6%, alcohol: 5%). In total we observe 13790 deaths among offenders, of which 4 906 are individuals with a criminal record that includes drug-or alcohol-related crimes.

**Table 1:** Categories in the sample.

	Total		Men		Women	
	N	Proportion died	N	Proportion died	N	Proportion died
Total	2 872 358	3,7	1 456 551	4,6	1 415 807	2,9
No criminal record	2 586 840	3,5	1 222 129	4,4	1 364 711	2,8
Imprisoned for use/possession of drugs (once)	2 920	11,9	2 654	11,9	266	11,3
Imprisoned for use/possession of drugs (2+ times)	6 890	14,9	6 128	15,2	762	12,9
Imprisoned for motor vehicle use under influence	3 332	17,3	3 164	17,4	168	14,3
Others imprisoned	28 864	8,1	26 819	8,1	2 045	8,1
Use/possession of drugs, not imprisoned (once)	9 283	5,7	6 870	6,0	2 413	4,9
Use/possession of drugs, not imprisoned (2+ times)	5 293	8,5	4 024	8,5	1 269	8,5
Motor vehicle use under influence, not imprisoned	403	15,8	347	14,9	56	21,7
Others with a criminal record	228 533	4,2	184 416	4,4	44 117	3,6

Criminal history 1992 through 1999 of Norwegian men and women aged 15–69.

The age-adjusted relative risks are presented in tables 2 and 3. These figures are from the regression analyses run separately for men and women, and we only report the parameters of interest (full model results are available from the authors on request). The first model shows that the risk of death for offenders relative to the control group (non-offenders) is 2.1 times higher for males and 2.5 times higher for females. These estimates lie in a similar range to differences between those with high and low educational or income levels [26], [43] but are lower than estimates for prisoners [12], [44]. The next model shows the results for the offender population split into those with and without an unconditional custodial sentence. Those with a custodial sentence have a higher relative death risk than other offenders (RR men: 4.9, women: 7.1).

**Table 2:** Age-adjusted relative risk estimates for relative mortality for Norwegian men.

	Model 1		Model 2		Model 3		Model 4	
	RR	CI	RR	CI	RR	CI	RR	CI
**Criminal history** (ref = no criminal record)								
Criminal record	2.03	(2.00–2.07)						
Criminal record, not imprisoned			1.91	(1.85–1.96)				
Imprisoned			4.85	(4.68–5.03)				
Imprisoned for motor vehicle use under influence (>2 times)					6.22	(5.63–6.86)	5.94	(5.38–6.56)
Imprisoned for use/possession of drugs (once)					8.84	(7.90–9.90)	8.54	(7.62–9.56)
Imprisoned for use/possession of drugs (>2 times)					12.13	(11.34–12.98)	11.60	(10.84–12.42)
Others imprisoned					3.38	(3.21–3.55)	3.25	(3.09–3.42)
Motor vehicle use under influence, not imprisoned					6.91	(5.16–9.25)	6.74	(5.03–9.02)
Use/possession of drugs, not imprisoned (once)					5.78	(5.23–6.39)	5.69	(5.15–6.28)
Use/possession of drugs, not imprisoned (>2 times)					8.59	(7.72–9.56)	8.38	(7.53–9.33)
Others with a criminal record					1.75	(1.70–1.80)	1.73	(1.68–1.78)
QIC	491,488		297,323		291,681		290,443	
QICu	491,488		297,323		291,682		290,444	

Note: M1 through M4 adjusted for age. M4 also adjusted for parents’ educational level.

Registered offences observed between 1 January 1992 and 31 December 1999, deaths observed from 1 January 2000 to 31 December 2008.

**Table 3:** Age–adjusted relative risk estimates for relative mortality for Norwegian women.

Men	Model 1		Model 2		Model 3		Model 4	
	RR	CI	RR	CI	RR	CI	RR	CI
**Criminal history** (ref = no criminal record)								
Criminal record	2.44	(2.34–2.55)						
Criminal record, not imprisoned			2.84	(2.68–3.02)				
Imprisoned			7.07	(6.22–8.04)				
Imprisoned for motor vehicle use under influence (>2 times)					8.33	(5.45–12.74)	7.96	(5.20–12.18)
Imprisoned for use/possession of drugs (once)					11.25	(7.83–16.17)	10.97	(7.64–15.75)
Imprisoned for use/possession of drugs (>2 times)					15.74	(12.86–19.28)	15.17	(12.40–18.56)
Others imprisoned					4.37	(3.59–5.31)	4.22	(3.47–5.13)
Motor vehicle use under influence, not imprisoned					8.45	(4.09–17.43)	8.49	(4.08–17.63)
Use/possession of drugs, not imprisoned (once)					7.97	(6.62–9.61)	7.78	(6.46–9.38)
Use/possession of drugs, not imprisoned (>2 times)					13.86	(11.43–16.79)	13.47	(11.11–16.33)
Others with a criminal record					2.45	(2.30–2.62)	2.43	(2.28–2.60)
QIC	327,735		155,638		154,170		154,028	
QICu	327,735		155,638		154,171		154,030	

Note: M1 through M4 adjusted for age. M4 also adjusted for parents’ educational level.

Registered offences observed between 1 January 1992 and 31 December 1999, deaths observed 1 January 2000 to 31 December 2008.

In model three, the offender group is further sub-divided into whether offenders have a record of any alcohol or drug-related offences, yielding the full set of categories presented in table 1. The risk of death for male offenders with a record of neither alcohol- and drug-related crimes nor any unconditional custodial sentence is 1.8 times higher than for non-offenders (RR = 2.5 for females). This is the largest group of offenders, and is only a little smaller than the initial estimate for all offenders in model 2. In accordance with our expectations, those with drug-related offences on their record have a far higher risk of death. Those with drug-use-related records who have once been imprisoned have very high mortality risk (RR men: 8.8, women: 11.3); particularly if they have been imprisoned multiple times (RR men: 12.1, women: 15.7), followed by those who have multiple drug record but no imprisonment (RR men: 8.6, women: 13.9) and one drug record but no imprisonment (RR men: 7.0, women: 10.8). Those with a record of once driving under the influence of substances do not have quite as high a death risk (males: 6.2 if imprisoned and 6.9 if not imprisoned, females: 8.3 if imprisoned and 8.5 if not imprisoned). Others with an unconditional custodial sentence have a relative risk of death of 3.4 (men) and 4.4 (women), while among those who have not been in prison, men have a relative risk of death of 1.8 and women 2.5.

The final model reports the RR, also adjusting for parental education as a proxy for social class. Although this reduces the elevated RR somewhat, it does not change the picture substantially. The largest reduction in RR was for those with a record of multiple imprisonment for drug-related crimes, which reduced the relative risk by about 0.5 (women: from 15.7 to 15.1, men: 12.1 to 11.6). The other adjustments were even smaller and certainly not significantly different from the estimates in model 3; thus social class has little or no explanatory power for offenders’ elevated death risk.

## Discussion

In a total population sample from Norway including all those with a criminal record, we find that the level of relative mortality of Norwegian criminal offenders is very high relative to the overall population. We find that death risks are particularly high for those who are subject to alcohol and drug offenses. Even though alcohol and drug dependency are common in the offender population and these have a very high relative risk of death, drug-dependency are not the only reasons for an elevated death risk among offenders. Even for offenders who have no record of drug-related offences and have not served a prison sentence, the risk of death is still almost twice that of the non-offender population.

The relative risk of death is very high among those with an unconditional custodial sentence and even higher among prisoners with a record of drug-related offences. This is true also for drug users who have not been imprisoned. It is of particular interest to note that the age-adjusted relative risk of death is consistently higher for female offenders than for male offenders, and this holds across all kinds of criminal records. It is also notable that these differences cannot be explained by socio-economic traits, even though criminal record and mortality are strongly correlated with these traits.

We find that the excess mortality of females with a criminal record, which is 1.3–1.7 times greater than that of men, depending on the criminal category. The lowest difference between the genders is found for those with non-addiction related offences, while the highest difference is observed for those having committed drug- and alcohol-related offences. About 20 times as many men as women go to prison in Norway, which could imply that women offenders are more negatively selected than men. Previous studies of female offenders show that they fare worse than men on a range of outcomes: once women become addicted or commit criminal offences this may increase the risk of being ostracised, of self-debilitating behaviour, depression, social isolation, taking up prostitution, being infected by sexually transmittable diseases including HIV/AIDS, as well as self-harming behaviour and suicide [45], [46].

Our results are also interesting for a rich welfare state like Norway, where criminal policy recognizes the need for rehabilitation, to reduce harm caused by imprisonment, and young offenders are often given a second chance [47]–[49]. Nevertheless, the relative mortality among offenders, particularly for those caught for substance use, tend to be high in Norway, also when compared to many other western countries [1], [3], [6], [9], [11], [13].
